# Reconstruction of temporal activity of microRNAs from gene expression data in breast cancer cell line

**DOI:** 10.1186/s12864-015-2260-3

**Published:** 2015-12-18

**Authors:** Naresh Doni Jayavelu, Nadav Bar

**Affiliations:** Department of Chemical Engineering, Norwegian University of Science and Technology, Trondheim, N7491 Norway

**Keywords:** Network component analysis, microRNAs, Breast cancer, Activity, Data decomposition, Cancer markers, EGFR signaling, Survival analysis, Kaplan-Meier plots

## Abstract

**Background:**

MicroRNAs (miRNAs) are small non-coding RNAs that regulate genes at the post-transcriptional level in spatiotemporal manner. Several miRNAs are identified as prognostic and diagnostic markers in many human cancers. Estimation of the temporal activities of the miRNAs is an important step in the way to understand the complex interactions of these important regulatory elements with transcription factors (TFs) and target genes (TGs). However, current research on miRNA activities excludes network dynamics from the studies, disregarding the important element of time in the regulatory network analysis.

**Results:**

In the current study, we combined experimentally verified miRNA-TG interactions with breast cancer microarray TG expression data to identify key miRNAs and compute their temporal activity using network component analysis (NCA). The computed activities showed that miRNAs were regulated in a time dependent manner. Our results allowed constructing a synergistic network of miRNAs using the computed miRNA activities and their shared regulation of TGs. We further extended this network by incorporating miRNA-TG, miRNA-TF, TF-miRNA and TF-TG regulations in the context of breast cancer. Our integrated network identified several miRNAs known to be involved in breast cancer regulation and revealed several novel miRNAs. Our further analysis detected substantial involvement of the miRNAs miR-324, miR-93, miR-615 and miR-1 in breast cancer, which was not known previously. Next, combining our integrated networks with functional annotation of differentially expressed genes resulted in new sub-networks. These sub-networks allowed us to identify the key miRNAs and their interactions with TFs and TGs of several biological processes involved in breast cancer. The identified markers are validated for their potential as prognostic markers for breast cancer through survival analysis.

**Conclusions:**

Our dynamical analysis of the miRNA interactions greatly helps to discover new network based markers, and is highly applicable (but not limited) to cancer research.

**Electronic supplementary material:**

The online version of this article (doi:10.1186/s12864-015-2260-3) contains supplementary material, which is available to authorized users.

## Background

Cell functions are exerted through gene regulation in response to external cues. MicroRNAs (miRNAs) and transcription factors (TFs) are key regulators in the gene regulation process [1, 2]. miRNAs are small (~22 nucleotides in length) non-coding RNAs that regulate gene expression post transcriptionally in a sequence-specific manner [3]. Many miRNAs are shown to be involved in cancer related biological processes, such as cell division, growth, development, apoptosis, proliferation and differentiation [4–7]. Therefore, constructing the miRNAs mediated gene regulation networks by utilizing gene expression data has become a regular practice in today’s miRNA research. However, all these studies of miRNA regulatory networks focused on static reconstruction of the miRNA regulatory activities. By doing so, they excluded the important element of time from the network analysis. However, since we know regulatory networks are dynamic (time dependent) by nature, important network information in those studies may have been lost.

Several studies applied statistical methods to investigate the role of miRNAs in gene regulatory networks. Madden et al identified key miRNAs associated with diseases through time–independent multivariate statistical analysis [8]. _ENREF_47Liang et al developed a web based tool to compute the microRNA activity from its TG expression data based on the negative regulatory relationship between miRNAs and TGs [9]. Mezlini et al developed a regression model to identify key miRNAs and their activity from TG expression and miRNA-TG network [10]. _ENREF_47_ENREF_44 The approach proposed by Cheng et al [11] computed a series of static miRNA activities using the differential expression values of the TGs at each time point. Although their approach appears to construct a time-series miRNA activity profiles, it considers each time point regardless of the expression levels in the other time points. Recently, Schulz et al extended the DREM (Dynamic Regulatory Events Miner) model to mirDREM to reconstruct the dynamic miRNA regulated interaction networks [12]. This model presents the list of significantly pivotal miRNAs and TFs at each time point. However, none of these methods computed the changes in miRNA activity with time.

Network component analysis (NCA) [13, 14] is a data decomposition approach that has been successfully employed in several species and in numerous research studies to compute the temporal activity profiles of TFs and construction of dynamic networks [14–28]. The method integrates temporal TG expression data and known network topology. _ENREF_24 In the current study, we exploited this approach for computing the temporal activities of the key miRNAs using only TG expression data (no miRNA data) and experimentally verified miRNA-TG relations. Using the NCA, we identified the key miRNAs, TFs and their activities in epidermal growth factor receptor (EGFR) signaling in breast cancer cells._ENREF_22_ENREF_23 We used the computed miRNAs temporal activities to identify co-regulating miRNAs (synergistic network) that show similar activity patterns and co-regulating common TGs, and validated these miRNAs with a literature study. Additionally, we built an integrated network of miRNAs, TFs and their TGs by retrieving miRNA-miRNA, miRNA-TG, TF-TG and TF-miRNA interactions from literature and combining these with the results of the NCA. With this approach, we identified several miRNAs that were known to be involved in regulation in breast cancer cells, and we revealed several novel miRNAs that are most likely to be involved in breast cancer, but were not known previously. These miRNAs can potentially serve as breast cancer markers.

## Results

**Fig. 1:**
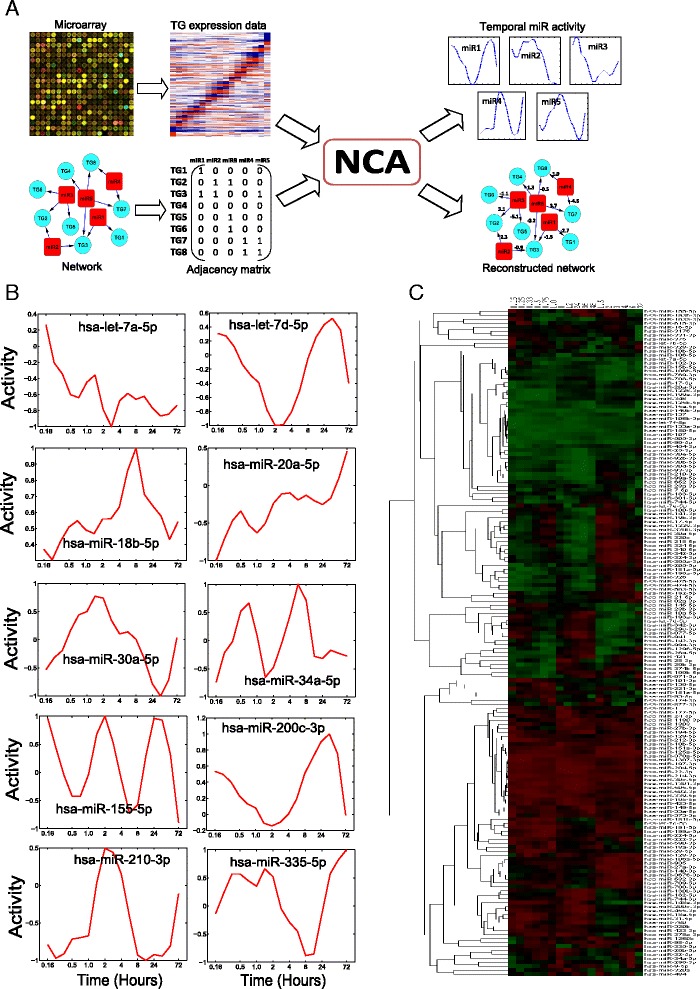
Schematic of the approach and computed miRNA activities: (**a**) NCA approach for reconstructing the temporal activities of miRNAs. **b** Reconstructed temporal activity profiles (normalized values) of selected breast cancer associated miRNAs. **c** Hierarchical clustering of miRNAs activities. Here, each row represents a target, and the column represents time point

Our approach for reconstructing the miRNA temporal activity from its TG expression using NCA is presented in Fig. 1a and complete details are described in the Methods section.

### Dynamics of miRNA activity

The activity profiles (normalized) of several miRNAs which are already known to be involved in breast cancer cells are presented in Fig. 1b. Although the miRNAs showed activity at all-time points, peak activity is demonstrated at 1 or 2 time points. The miRNA let-7a-5p displayed increasing repressing activity with time. This miRNA is known to be a tumor suppressor regulating many genes that inhibit cell migration in breast cancer [29]. The miRNA miR-18b-5p is also involved in breast cancer, regulating genes involved in cell migration and metastasis [30]. This miRNA showed peak activation at 8 h in the current study. The miRNA let-7d-5p showed a peak repressing activity around 2 h after EGF treatment and it is aberrantly expressed in breast cancer cells in previous study [31]. The miRNA miR-20a-5p displayed a peak repressing activity at 10 min and this miRNA also involved in previous breast cancer studies [32]. Yu et al showed that miR-20a-5p and miR-17-5p suppressed the breast cancer cell proliferation by negatively regulating the gene cyclin D1 [32]. The miRNA miR-30a-5p is identified to be a novel prognostic marker in breast cancer in several past studies [33–35] and it showed a peak repression very late (around 36 h) in the current study. The miRNA miR-200c-3p also displayed a very late activation at 36 h and it is involved in regulating epithelial to mesenchymal transition (EMT) by targeting the genes ZEB1 and SIP1 in breast cancer in response to transforming growth factor (TGF) [36]. The miRNA miR-155-5p demonstrated periodic peak activations at 10 min, 2 and 24 h. This miRNA is also known to be involved in the previous breast cancer studies with roles in cell survival, growth and chemosensitivity [37, 38]. The miRNA miR-210-3p exhibited peak repression activities very early at 15 min and late during 8–36 h time period and this miRNA has been identified as prognostic marker in breast cancer [39]. Next, we used hierarchical clustering to explore groups of miRNAs with similar activity profiles (Fig. 1c). We found two distinctive groups of miRNAs that activates or repress at all time, and two smaller groups of miRNAs that alternate between activation and repression.

**Fig. 2:**
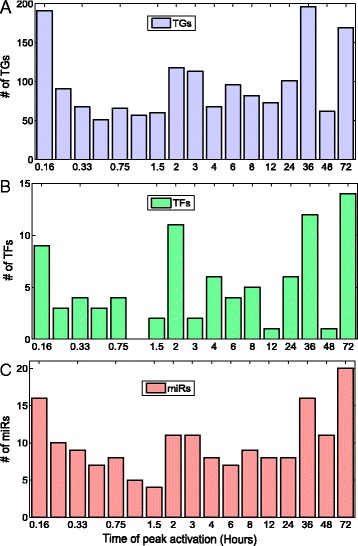
EGF dynamic regulation: The number of active TGs, TFs and miRNAs at each time point is presented. (**a**) TGs (**b**) TFs (**c**) miRNAs. The active TGs, TFs and miRNAs are defined at each time point based on peak expression or activity at that time point

The computed activities demonstrated that EGF activated microRNAs in time dependent manner. To further understand the timely cascade of regulation of EGF in breast cancer, we studied the time points of peak activation of the TGs, (from differential expression data) the TFs and the miRNAs, the last two were computed from NCA (Fig. 2). This analysis revealed that EGFR signaling is a highly dynamic process, and the regulation operates in cascades, activating groups of TGs, miRNAs and TFs in a timely manner. The larger effects of EGF stimulation on TGs are observed around 10 min, 2 h 36 h and 72 h. Interestingly, for the regulators (miRNAs and TFs) also larger effects are observed at the same time points respectively.

### miRNA-miRNA synergistic network

**Fig. 3:**
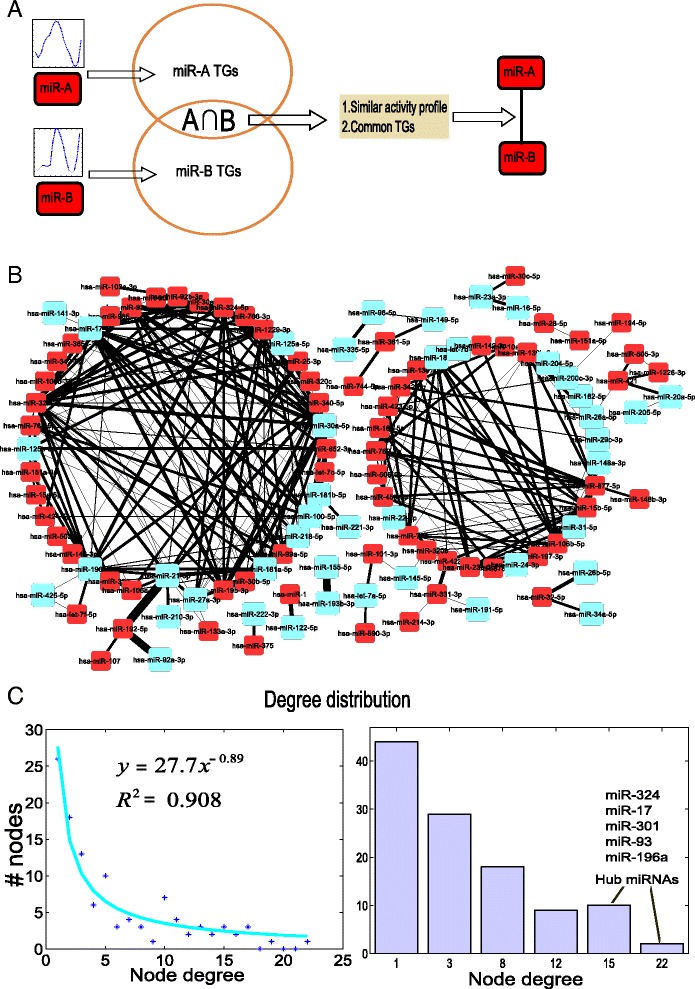
Synergistic network of miRNAs: (**a**) Schematic of miRNA-miRNA synergistic network construction. **b** Predicted synergistic interaction pairs of miRNAs in the form of network in breast cancer. Here cyan squares represent breast cancer associated miRNAs in previous studies and red squares represent new miRNAs with no previous involvement in breast cancer studies. **c** Degree distribution. Right panel: scatter plot of the node degree (number of connections to a specific miRNA) vs. the number of nodes (miRNAs) revealed the power law degree distribution. Left panel: Distribution of miRNA as a function of their node number. We found several hub miRNAs with large number of connections

Synergistic interactions between miRNAs are a key to understand the complex mechanisms of cancers, several miRNAs are usually found together in a particular cancer or disease. In the present study, a miRNA-miRNA synergistic network (MMSN) was constructed based on the computed temporal activities and shared co-regulating TGs (Fig. 3a). In this constructed network, each node represents the miRNA and edges represent similar activity profile (Pearson correlations > 0.7) and at least 3 shared TGs. Thus, the constructed network included 112 miRNAs and 314 interactions between the miRNAs (Fig. 3b, Additional file 1). The network followed a power law degree distribution which is a typical biological network property (Fig. 3c). The network is highly interconnected with a clustering coefficient of 0.356 and a mean connectivity of 5.6. 25 % of miRNAs have at least 10 synergistic interactions with other miRNAs. 38 % of the miRNAs in this network were already experimentally verified to be involved in breast cancer studies (according to miRcancer, miR2Disease databases and manual curation [40, 41]). The miRNAs miR-324-3p, miR-17-5p, miR-30a-5p, miR-93-3p and miR-196a-5p were densely connected, each having more than 17 synergistic interactions with other miRNAs in the network. Furthermore, these miRNAs are interacting with at least 5 known breast cancer associated miRNAs in the network. Of these, miR-17-5p, miR-30a-5p and miR-196a-5p were already known to be involved in breast cancer. To further strengthen our prediction of miRNA-miRNA interactions, we compared the predictions from this study with previously reported data. For this purpose, we downloaded complete miRNA-miRNA regulation data of Sengupta et al [42] and found that more than 80 % (258 out of 314) overlapping interactions between these two studies (Additional file 2). We stress that although we found a large overlap between the two studies, the interactions from Sengupta et al [42] are purely computational, just as our’s do, but are independent of ours, taken with a completely different approach. In addition to this, we evaluated the synergy of miRNAs in the current network with randomly generated 100 miRNA-miRNA networks keeping the same node degrees as the original network. The mean value (0.114) for clustering coefficient of the random networks is significantly (*P*-value < 1e-10) lower than the value (0.356) of the original network.

### Analysis of the integrated network in breast cancer

One of the main objectives of this study is to determine the core regulators of EGFR signaling in breast cancer cells and understand their role in breast cancer. To achieve this goal, we constructed an integrated network of miRNAs, TFs and TGs (Methods, Additional files 1, 2, 3, 4, 5, 6 and 7). The final integrated network followed a power law degree distribution (The network is provided as a cytoscape file (.cys) in Additional file 3). The network includes 168 miRNAs, 328 TFs and 1072 TGs and 20534 interactions. In this network, we find that 87 % of the TFs have at least 3 or more connections. Similarly, 89 % (150/168) of the miRNAs regulate at least five TGs and 82 % of TGs are targeted by five or more regulators including both miRNA and TFs. Thus, the integrated regulatory network is complex in terms of targets multiplicity and miRNAs co-operativity in the regulation of TGs. The in-degree and out-degree distributions of the network follow a power-law degree distribution with slopes of −1.9 and −4.66 respectively. This indicates that the network is not random but a complex biological network with organized structure [43]. We hypotheses that regulating elements with large number of connections (node degree, whether they are miRNAs or TFs), can be considered as ‘core’ regulators [43]. In order to identify these core regulators, we focused on hub nodes with the largest number of interactions in the network. The identified top 20 hub miRNAs and TFs are presented in Table 1 and Table 2 respectively. Of these, 14 miRNAs were already known to be involved in breast cancer cells and six were unexplored yet. There were 11 TFs known to be involved in breast cancer, and 9 that were yet to be studied (Table 2).

**Table 1:** The top 20 miRNAs with highest degree in breast cancer integrated regulatory network. The degree of a node is the sum of in-coming and out-going connections with other nodes in the network. PMIDs denote the ‘pubmed’ identification numbers

Rank	miR Name	Degree	Breast cancer related	PMIDs
1	hsa-miR-335-5p	220	Yes	18185580
2	hsa-miR-124-3p	185	Yes	22333974, 22085528
3	hsa-miR-26b-5p	174	Yes	23374284, 21510944
4	hsa-miR-16-5p	137	Yes	22583478
5	hsa-let-7b-5p	114	Yes	23339187
6	hsa-miR-615-3p	110	No	--
7	hsa-miR-155-5p	107	Yes	23372341, 16103053
8	hsa-miR-92a-3p	105	Yes	23052693
9	hsa-miR-1	96	No	--
10	hsa-miR-21-5p	90	Yes	23052693, 19419954
11	hsa-miR-484	88	No	--
12	hsa-miR-30a-5p	86	Yes	22476851, 23389917
13	hsa-miR-193b-3p	82	Yes	19701247
14	hsa-miR-34a-5p	79	Yes	23032974
15	hsa-miR-17-5p	78	Yes	16940181, 20505989
16	hsa-miR-192-5p	77	No	--
17	hsa-miR-324-5p	73	No	--
18	hsa-miR-98-5p	72	Yes	18812439
19	hsa-miR-324-3p	71	No	--
20	hsa-miR-125b-5p	64	Yes	22693547, 21444677

**Table 2:** The top 20 TFs or TGs with highest degree in breast cancer integrated regulatory network. The degree of a node is the sum of in-coming and out-going connections with other nodes in the network. PMIDs denote the ‘pubmed’ identification numbers

Rank	TF Name	Degree	Breast cancer related	PMIDs
1	SP1	371	Yes	19812674
2	TCF12	343	Yes	20525248
3	SP4	337	No	--
4	SP3	309	No	--
5	SP2	259	Yes	Breast cancer database (www.breastcancerdatabase.org)
6	MYOD1	222	Yes	20525248
7	JUN	215	Yes	20525248
8	ERG	211	No	--
9	MYF6	199	No	--
10	ETV7	189	Yes	20525248
11	ARID5B	187	No	--
12	MYF5	183	No	--
13	EGR2	182	Yes	20525248
14	MYOG	182	No	--
15	ETS1	181	Yes	20668451, 20525248
16	ASCL1	180	No	--
17	MYC	178	Yes	20525248
18	ETS2	176	Yes	Breast cancer database (www.breastcancerdatabase.org)
19	TCF3	174	No	--
20	ELF2	173	No	--

### Functional annotation and pathway analyses of differentially expressed TGs

To increase our understanding on the role of EGF in breast cancer, we performed functional annotation of the TGs and TFs in the integrated network using functional annotation tool DAVID (Methods). We found that pathways in cancer, TGF-beta signaling pathway, the MAPK signaling pathway, the Wnt signaling pathway, the cell cycle, Notch signaling pathway, melanogenesis, the ErbB signaling pathway and several cancers were all statistically significant affected by the differentially expressed genes in breast cancer cells (Table 3).

**Table 3:** Statistically significant biological pathways affected by differentially expressed TGs in breast cancer cells identified from the KEGG database using DAVID

KEGG ID	Description	# Genes	*P*-Value
hsa05200	Pathways in cancer	69	5.51E-08
hsa04350	TGF-beta signaling pathway	24	3.61E-05
hsa04010	MAPK signaling pathway	51	6.72E-05
hsa04310	Wnt signaling pathway	31	7.22E-04
hsa04110	Cell cycle	25	0.003827
hsa04330	Notch signaling pathway	11	0.027579
hsa04662	B cell receptor signaling pathway	15	0.030166
hsa04910	Insulin signaling pathway	23	0.034331
hsa04660	T cell receptor signaling pathway	19	0.043201
hsa04012	ErbB signaling pathway	16	0.047711

The significantly enriched biological terms that were identified from the PANTHER database include PDGF signaling pathway, PI3 kinase pathway, JAK/STAT signaling pathway, apoptosis pathway and several overlapping pathways that are identified from KEGG database (Full list is provided in Additional files 4 and 5). The involvement of these pathways in human breast cancers has been described in previous studies [44–47]. This analysis facilitated the identification of previously known and newly discovered pathways in breast cancer. Positive and negative regulation of transcription and gene expression, regulation of transcription factor activity, regulation of cell differentiation, proliferation, migration, apoptosis, morphogenesis, angiogenesis and regulation of signal transduction are enriched biological processes (Table 4).

**Table 4:** Statistically significant biological processes affected by differentially expressed TGs in breast cancer cells identified using DAVID

GO ID	Description	# Genes	*P*-Value
GO:0010628	positive regulation of gene expression	220	2.59E-79
GO:0045941	positive regulation of transcription	215	4.94E-78
GO:0051173	positive regulation of nitrogen compound metabolic process	229	1.65E-76
GO:0016481	negative regulation of transcription	122	3.60E-26
GO:0010629	negative regulation of gene expression	129	5.17E-26
GO:0045596	negative regulation of cell differentiation	66	1.18E-17
GO:0045597	positive regulation of cell differentiation	64	5.79E-15
GO:0042981	regulation of apoptosis	139	4.34E-12
GO:0008285	negative regulation of cell proliferation	72	3.28E-09
GO:0008284	positive regulation of cell proliferation	73	4.58E-07
GO:0051090	regulation of transcription factor activity	28	9.89E-07
GO:0045787	positive regulation of cell cycle	16	2.38E-04
GO:0009966	regulation of signal transduction	115	4.86E-04
GO:0010608	posttranscriptional regulation of gene expression	37	4.93E-04
GO:0000902	cell morphogenesis	55	5.06E-04
GO:0030334	regulation of cell migration	31	7.23E-04
GO:0001525	angiogenesis	28	8.51E-04

**Fig. 4:**
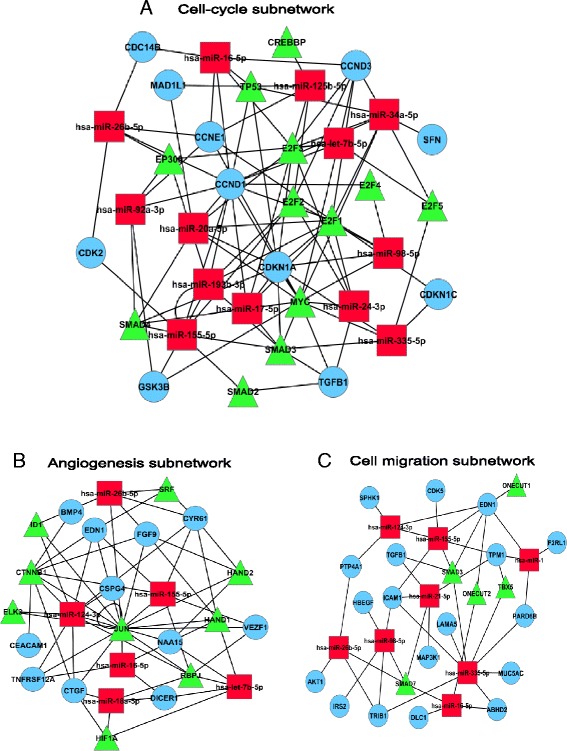
Sub-networks of biological processes: (**a**) Cell-cycle sub-network. **b** Angiogenesis sub-network. **c** Cell migration sub-network

Next, we combined the pathways and gene ontology (GO) results with the integrated network and extracted significantly enriched biological process specific sub-networks (Fig. 4). For instance, Cell-cycle sub-network is mainly regulated by E2F family of TFs (E2F1, E2F2, E2F4, and E2F5), SMAD family of TFs (SMAD2, SMAD3, SMAD4) and miRNAs has-miR-335-5p, has-miR-26b-5p and has-miR16-5p. Many of these regulators are involved in cell-cycle control. In the similar manner, we constructed the angiogenesis and cell migration sub-networks.

## Discussion

Understanding regulation and precise control of gene expression in higher organisms is a complex process, and miRNAs and TFs are two key regulators of this process. In the current study, we used the well-studied NCA approach to compute the temporal activities of the TFs, and for the first time, for the miRNAs as well. Although several previous studies demonstrated the construction of miRNA mediated gene networks, their approaches required the expression data of both miRNAs and TGs. In contrast, our reconstruction approach needs only expression data of TGs. With the publicly available large volumes of the microarray and RNA-sequencing (RNA-seq) TG expression data and experimentally verified miRNA-TG data, the NCA approach may serve as a powerful tool to study and understand the miRNA mediated gene regulation. With the computed temporal activities and gene expression data, we are able to identify the time specific active miRNAs, TFs and TGs. This analysis resulted in the identification of EGF stimulation’s dominant response at selective time points. Another interesting observation from this analysis is that the number of activated TGs are strongly correlated with their active regulators, TFs (Pearson correlation = 0.815) and miRNAs (Pearson correlation = 0.867) over entire time period.

We constructed the miRNA-miRNA synergistic network based on similar temporal activity of miRNAs and their shared TGs. There are several past studies constructed miRNA-miRNA networks but they mostly are based on combinations of shared TGs of miRNA pair, enriched in same gene ontology term, sequence, secondary structure and shared pathways [48–51]. However, none of these studies were used the temporal information knowing that miRNA-TG regulation is highly dynamic. Therefore, the synergistic network constructed in this study is one of the first attempts to incorporate temporal information. This network not only captured synergistic interactions between miRNAs but also identified novel miRNA regulators in breast cancer.

To understand the miRNA regulation more comprehensively, we further extended this synergistic network with TGs and TFs. Further examination of network identified hub miRNAs (hsa-miR-335-5p, hsa-miR-124-3p, hsa-miR-26b-5p, and hsa-miR-16-5p) and TFs (SP1, TCF12, JUN, MYOD1). Most of these hubs are either well-known regulators or are reported to play key roles in breast cancer. For instance, the miRNA miR-335-5p, the top hub node in the network is already known to be a key regulator in suppressing breast cancer metastasis and migration through regulation of targets SOX4 and TNC [52]. The miR-124-3p was shown to be a novel tumor suppressor and a co-regulating EGFR driven cell cycle protein, inhibiting proliferation in breast cancer [53]. The miRNA miR-26b-5p was also shown to be a potential therapeutic target for breast cancer. This miRNA inhibits the cell proliferation by regulating the target PTGS2 [54]. The synthetic growth hormone progestin down regulated the miR-16-5p and cyclin E was identified as one of its targets in breast cancer [55]. Furthermore, this miRNA inhibited the growth of progestin treated breast cancer cells and thus its role as tumor suppressor. The miRNA let-7b-5p was also shown to have a tumor suppressor role in breast cancer patients with lymph node metastasis, by repressing the expressions of the genes PAK1, DIAPH2, RDX and ITGB8 [29]. The miRNA miR-193b-3p was shown to be an important marker in clinical metastasis of human breast cancer cells, which potentially up-regulates the expression of uPA [56]. In addition to those hub miRNAs, we found hub miRNAs with no previous association in breast cancer, including miR-615-3p, miR-1, miR-484, miR-192-5p and miR-324-5p. We suggest that the novel miRNAs found from our integrated network have potential therapeutic outcomes in breast cancer and should be further explored. Similarly, the top hub TFs we found in the integrated network such as SP1, SP2, TCF12, MYC, JUN and EGR2, were also well-known regulators in breast cancer. Yang et al showed that SP1 and HSF1 play an important role in the regulation of FUT4 (Fucosyltransferase IV), which is associated with breast cancer epithelial cell proliferation [57]. Zhang et al identified that oncoprotein HBXIP activates the gene PDGFB through transcription factor SP1, to promote proliferation in breast cancer cells [58]. Chen et al showed that JUN miR-21 activates Bcl-2 expression and thus promotes chemo resistance in triple negative breast cancer cells [59]. The TF ETS1 promotes proliferation, migration and invasion through stimulation of estrogen receptor alpha (ERα). Verschoor et al showed the ETS1 involvement in energy metabolism and oxidative stress in breast and ovarian cancers [60].

**Fig. 5:**
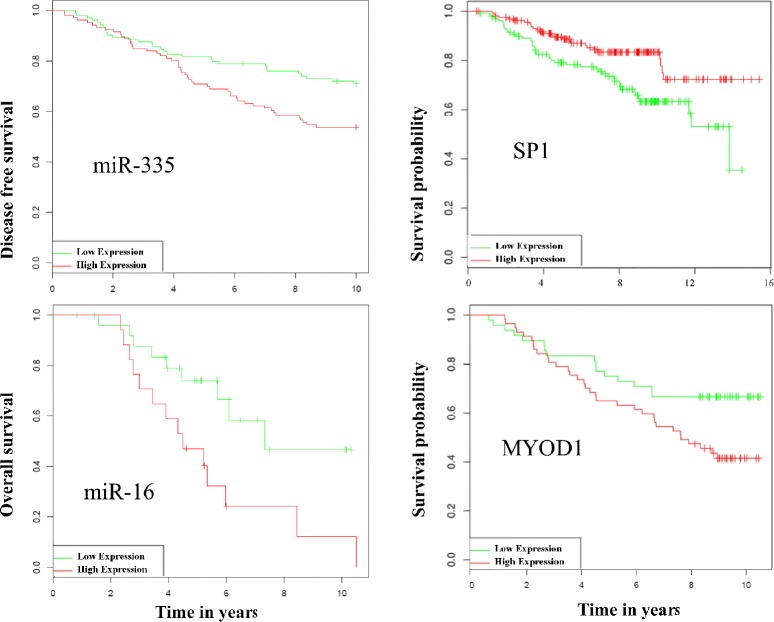
Survival analysis: The Kaplan-Meier survival plots for the selected miRNAs (left panel) and TFs (right panel). The disease free survival refers to the probability of patients free from the disease (here breast cancer) and the event is relapse rather than death. The overall survival rates are usually based on the death. The overall survival is also referring to the survival probability

To further validate our identified markers both miRNAs and TFs, we performed survival analysis with the publicly available clinical data to uncover their role in breast cancer survival outcome (see Methods for details). Figure 5 presents the Kaplan-Meier plots for the miRNAs and TFs, selected from Tables 1 and 2, respectively (see Additional file 5 for the remaining miRNAs and TFs). The patients with high expression of miR-335 and miR-16 had significantly better survival rates compared to patients with low expression. Similar findings are observed with the TFs SP1 and MYOD1. We suggest that these miRNAs and TFs can potentially serve as positive prognostic markers in breast cancer. We note that although the majority of the markers presented in this study (Tables 1 and 2) were identified as markers with potentially better survival rates, few markers did not demonstrated in the survival analysis any better performance. For instance, TCF12 which was identified as a marker in this study did not show better survival (see Additional file 6A).

We further analyzed the TGs and TFs from integrated network to find the common KEGG pathways and Gene Ontology (GO) biological process terms they regulated. Several previous studies showed that EGFR signaling is one of the potentially targeted pathways for identifying anticancer drugs and treatment strategies for various cancers [61, 62]. The involvement of Wnt signaling pathway in breast cancers have been described previously. Schlange et al had shown that autocrine Wnt signaling controls proliferation and tumor growth through activation of canonical Wnt pathway and EGFR transactivation [63]. Loh et al had shown the important role of this pathway in inhibiting the effects of Tamoxifen in tumor growth [64]. TGF-beta signaling pathway is also widely studied to identify therapeutic drug targets in many metastatic cancers including breast cancer as this pathway plays a key role in regulating tumor invasion and metastasis [65–67]. Another significantly enriched term was apoptosis. Dysregulation of apoptosis was shown to play key roles in breast cancer [68]. This finding explains the role of EGF as a potential therapeutic target in breast cancers. The MAPK pathway is the central part of the signal transduction initiated by EGF that controls the cellular processes of proliferation and differentiation. This pathway was also highly enriched in the current study and has been widely targeted to find diagnostic and prognostic markers of breast cancer [69]. Although this analysis identified the ErbB as a significantly enriched pathway (as the gene expression data set is obtained from ErbB signaling), to our surprise only 16 out of 1072 differentially expressed TGs were known to be associated with ErbB. This may indicate that our current study identified several new TGs associated with this signaling.

There are few limitations in the current approach used in this study. Firstly, we used only the experimentally verified miRNA-TG regulations from miRTarBase database. This database contains the regulation data retrieved from heterogeneous systems and it may not be accurate for a specific system. Secondly, the NCA approach has very strict criteria on network structure (miRNA-TG, TF-TG) and might have lost few key miRNAs, TFs and their TGs. Thirdly, in spite of the fact that potential prognostic markers for breast cancer in this study were predicted using computational approach only, the validations were based on Kaplan-Meier survival analysis conducted with heterogeneous data sources from clinical trials.

## Methods

### Data preprocessing

The gene expression data used in this study were obtained by measuring the response of MCF7 breast cancer cells treated with epidermal growth factor (EGF) at 17 time points over a time period of 72 h [70]. The original gene expression data was downloaded from the GEO database with accession number GSE13009. We applied loess normalization within time points and quantile normalization across time points. The expression values were averaged over two replicate measurements. We computed statistical significance, *P*-values based on t-tests by comparing control versus treatment samples at each time point to identify differentially expressed genes (DEGs). The DEGs with a fold change > 1.5 and *P*-value < 0.05 at least at two time points were selected for further analysis. To reduce the noise and to smooth the data, we used Fourier transform functions to fit the time-series data [71]. The initial networks were defined using experimentally verified miRNAs, TFs and its interactions with TGs from databases. All the computations were performed using bioinformatics toolbox in MATLAB.

### miRNA-TG interactions

Although several databases are available for predicting miRNA-TG interactions, we chose miRTarBase because it contains manually curated and experimentally verified regulations [72]. We downloaded the regulation data as an adjacency list, which was used in NCA analysis to predict the temporal dynamic activity of miRNAs.

### TF-TG interactions

We collected the experimentally verified TF-TG regulations from TFacts [73], a database containing 6401 experimentally validated regulations between 2720 TGs and 330 TFs. This database includes integrated information from different resources, such as TRED, TRDD, PAZAR NFIregulomeDB and their own experimental predictions. In addition, we retrieved TF-TG interactions from the Chip-X experiments of Transcriptome Browser [74]. This list includes 312 TFs, 13133 TGs and the 173156 interactions among them.

### Network component analysis (NCA)

Network component analysis (NCA) is a computational method for reconstructing hidden regulatory signals (miRNAs activity or TFs activity) from gene expression data with known connectivity information in terms of matrix decomposition [13]. The NCA method can be represented in matrix form as follows:

1 $$ \left[E\right]=\left[C\right]\left[T\right] $$

where the matrix [*E*] represents the expression values of genes at various time points, the matrix [*C*] is the control strength of each miRNA on a target gene (TG), and the matrix [*T*] represents the activities of all of the miRNAs. The dimensions of [*E*], [*C*] and [*T*] are N X M, N X L and L X M, respectively. Where, N is the number of TGs, M is the number of time points or measurement conditions, and L is the number of miRNAs or TFs.

Based on above formulation, the decomposition of [*E*] into [*C*] and [*T*] can be achieved by minimizing the following objective function:

2 $$ \min \parallel \left(\left[E\right]-\left[C\right]\left[T\right]\right)\parallel $$

$$ \mathrm{s}.\mathrm{t}.\ \mathrm{C}\in {\mathrm{Z}}_0 $$

where *Z*_0_ is the initial connectivity pattern. [*C*] and [*T*] are estimated using a two-step least-squares algorithm and are normalized through a nonsingular matrix [*S*] according to

3 $$ \left[E\right]=\left[C\right]\left[T\right]=\left[C\right]\left[S\right]\left[{S}^{-1}\right]\left[T\right] $$

To guarantee the uniqueness of the solution for equation (3) up to a scaling factor, certain criteria, termed NCA criteria, must be satisfied:

- The connectivity matrix [C] must have full-column rank
- When a node in the regulatory layer is removed along with all of the output nodes connected to it, the resulting network must be characterized by a connectivity matrix that still has full-column rank
- The [T] matrix must have full row rank

Using NCA as the reconstruction method, we predicted significant miRNAs, TFs and their temporal activity profiles. The NCA toolbox can be downloaded from here (http://www.seas.ucla.edu/~liaoj/downloads.html).

### Integrated approach

**Fig. 6:**
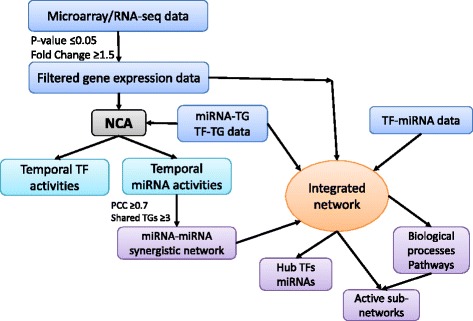
Schematic of integrated approach: The approach involves the pre-processing of the gene expression data, computing the temporal activities of miRNAs, TFs using NCA and construction of miRNA-miRNA synergistic network, integrated network and identification of significantly enriched biological processes and pathways. PCC stands for Pearson correlation coefficient, TF for transcription factor, TG for target gene and miRNA for micro RNA

Our integrated approach to study the miRNAs role in the gene regulation networks is composed of several phases (see Fig. 6 and Figs. 1a, 3a). Firstly, we downloaded the gene expression data, pre-processed and combined with connectivity data, run NCA to reconstruct temporal miRNA and TF activities. We clustered the miRNAs that exhibit similar temporal activity patterns and constructed the miRNA-miRNA synergistic network. We then constructed an integrated network by applying the NCA procedure using the differentially expressed genes (DEGs) data that we filtered, and the retrieved TF-TG topology (from the database TFacts and Transcriptome Browser), miR-TG (from the database miRTarBase), and TF-miR (from TransmiR). The interactions in all these databases were experimentally verified interactions. We then extended the resulting network with the predicted synergistic interactions of miRNAs. The detailed description and original sources are provided in the Additional file 7.

### The miRNA-miRNA networks

We computed the pairwise Pearson correlation coefficient between reconstructed activity profiles of all the miRNAs and the number of common TGs between each pair of miRNAs. We assumed a synergistic interaction between a pair of miRNAs if the correlation is greater than 0.7 and common TGs are greater than 3. The constructed network has 112 miRNAs and 314 synergistic interactions between them. Schematic of the approach is presented in Fig. 3.

The random networks for the comparison purposes are generated in Cytoscape software using ‘Randomnetworks’ plugin. These networks are created keeping the number of nodes and connections same as the original network.

### Pathway and biological processes

We used DAVID (Database for Annotation, Visualization and Integrated Discovery) with the default settings to find statistically enriched biological pathways. Information related to the pathways was identified from DAVID [75, 76]. DAVID is a comprehensive set of functional annotation tools for investigators to understand the biological meaning behind a large list of genes. DAVID uses the biological information retrieved from various resources and databases. For instance, information related to pathways is retrieved from KEGG (Kyoto Encyclopedia of Genes and Genomes), PANTHER, BioCarta and REACTOME pathway databases. Pathways and biological processes that had at least 10 DEG members and a *P*-value < 0.001 were considered significant. *P*-values are computed using modified Fisher’s exact test based on hyper geometric distribution.

The networks are created using the Cytoscape software tool [77]. All statistical calculations, NCA and clustering were done in Matlab, Mathworks.

### Survival analysis

We conducted survival analysis of miRNAs and TFs using the tools ‘*MIRUMIR*’ [78] and ‘*PPISURV*’ [79] respectively, both developed by Antonov AV et al. These tools integrate publicly available clinical data such as the GEO repository. Briefly, these tools utilize the rank information of expression profiles of miRNAs and TFs. Patients are divided into low and high expression groups, based on the average expression of the selected miRNAs or TFs. Then, the two distinguished groups of patients along with their survival information are used to identify any significant statistical differences in survival outcome using the statistical packages in R program. The survival outcomes are represented through Kaplan-Meier plots using R. The information about the clinical data source for survival analysis for miRNAs and TFs are provided in Additional file 6B.

## Conclusion

The analytical method we presented here was able to predict the involvement of several key miRNA regulators in processes related to breast cancer. It has also allowed us to explore the role of these regulators in the network and their interactions with TGs and TFs. We demonstrated that this dynamic miRNA-TF network analysis identifies regulation pathways, processes and connections that significantly involved in breast cancer. Furthermore, the identified markers are validated for their potential as prognostic markers for breast cancer though publicly available clinical data and survival analysis. We propose that this analysis can be applied to assist understanding miRNA regulation in other systems as well, suggesting individual miRNAs and entire pathways as target for cancer research.

## Additional files

Additional file 1:
**The complete list of predicted synergistic interactions of miRNAs is provided as a text file.** (ZIP 710 kb) Additional file 2:
**The overlapping synergistic interactions of miRNAs in this study and Sengupta et al study is provided as a tab delimited file (.txt).** (DOC 21 kb) Additional file 3:
**The integrated network as a cytoscape file (.cys file) is provided, which can be opened locally by a reader for interactive exploration.** (DOC 21 kb) Additional file 4:
**Statistically significant biological pathways affected by differentially expressed TGs in breast cancer cells identified from the PANTHER database is provided in a text file.** (DOC 21 kb) Additional file 5:
**Statistically significant biological pathways affected by differentially expressed TGs in breast cancer cells identified from the REACTOME database is provided in a text file.** (DOC 21 kb) Additional file 6:
**A: The Kaplan-Meier plots for the miRNAs and TFs.** B: The information about the clinical data source for survival analyses for miRNAs and TFs are provided in excel sheet. (DOC 21 kb) Additional file 7:
**The complete details of the integrated procedure with original sources are provided in a pdf file.** (DOC 21 kb)
