# Supplementary figures and images for: Reconstruction of temporal activity of microRNAs from gene expression data in breast cancer cell line
Source: BMC Genomics. 2015 Dec 18;16:1077. doi: 10.1186/s12864-015-2260-3 (PMC4712512; doi:10.1186/s12864-015-2260-3)

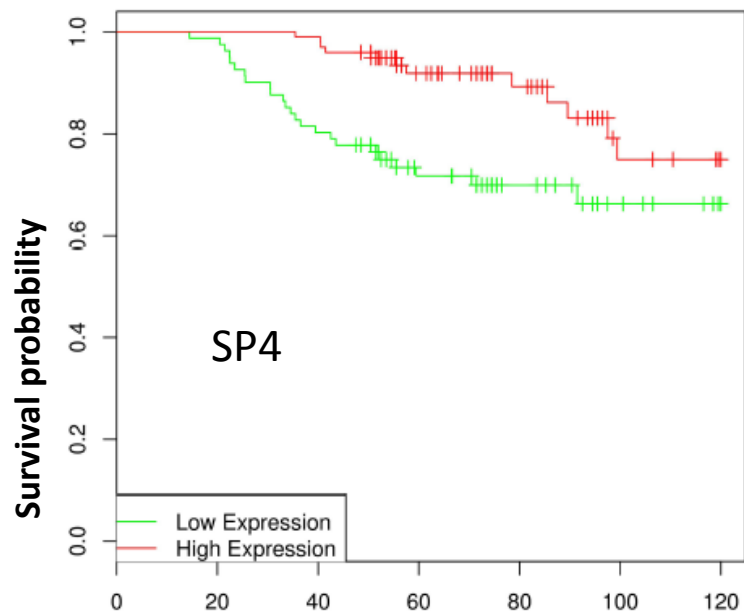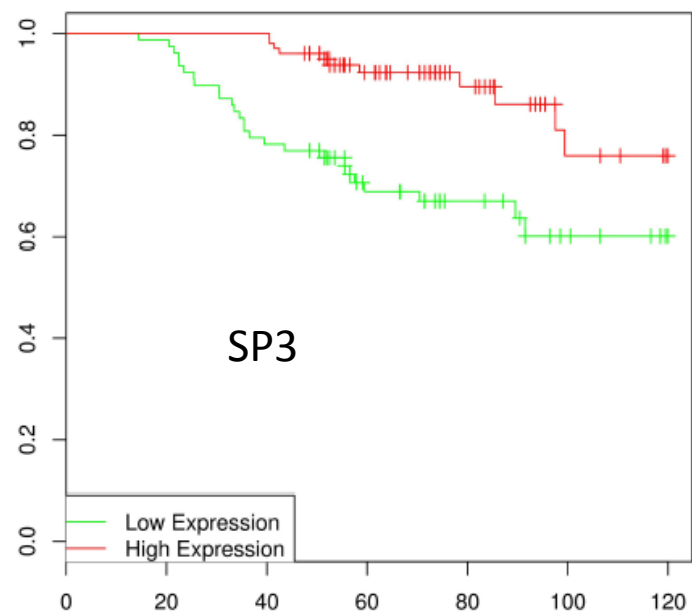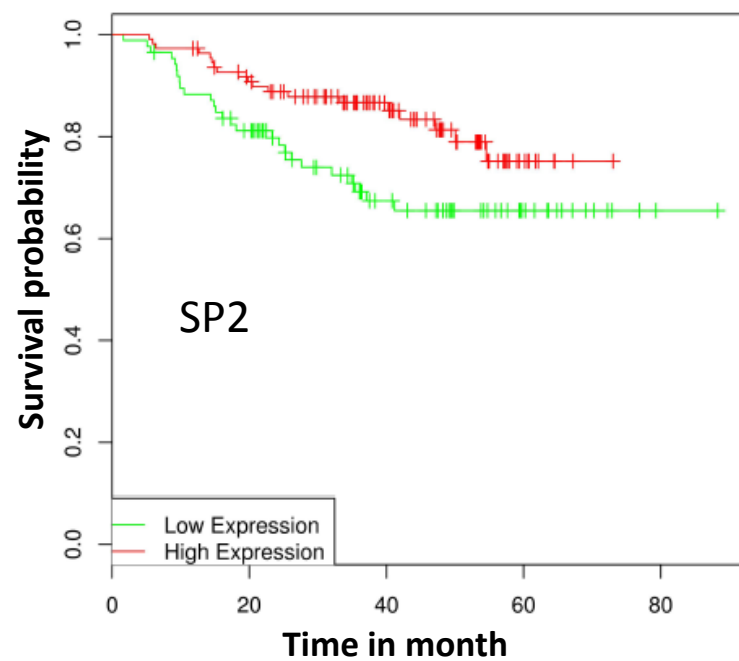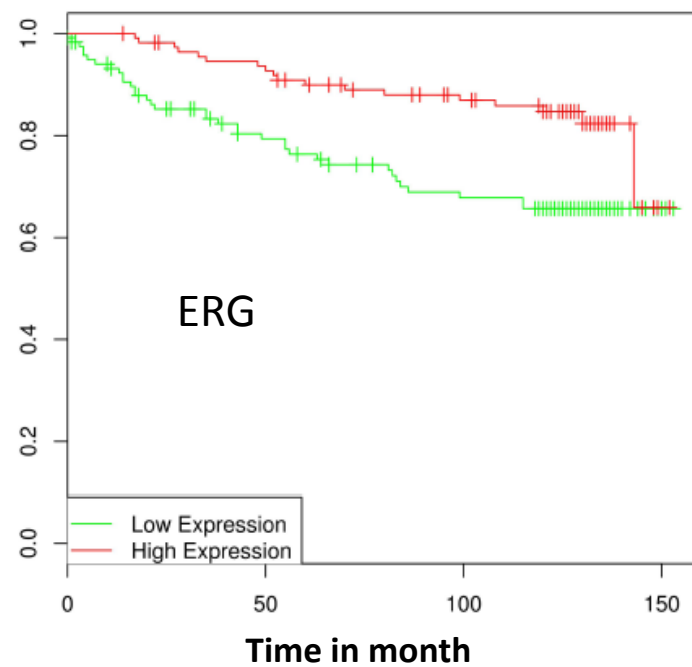

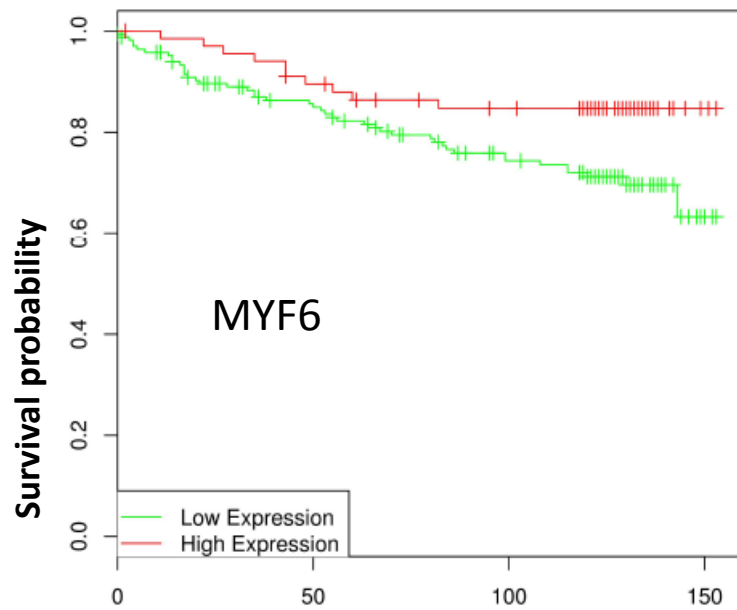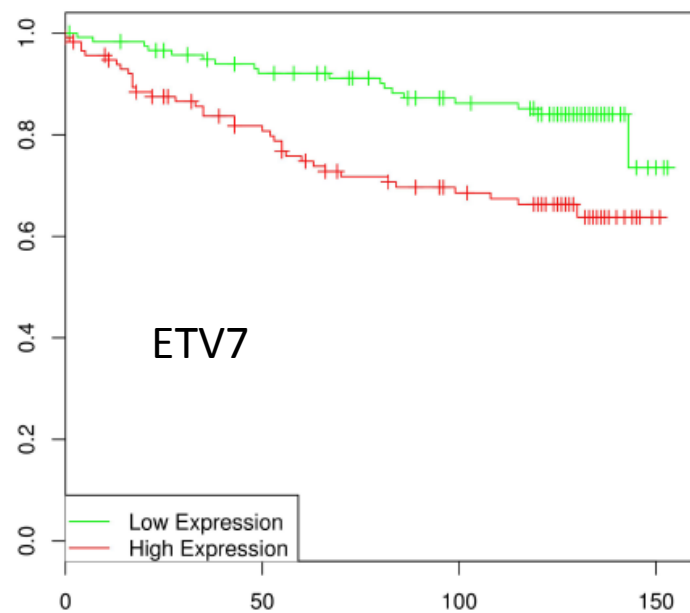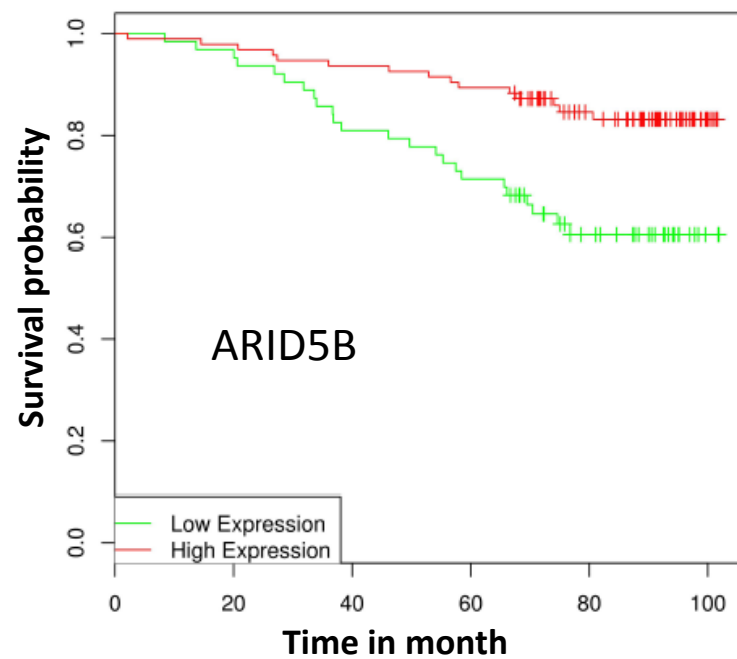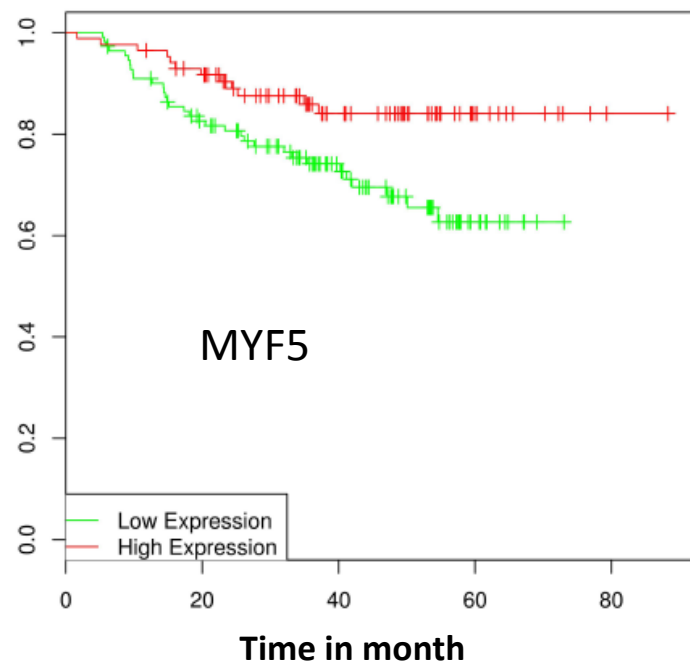

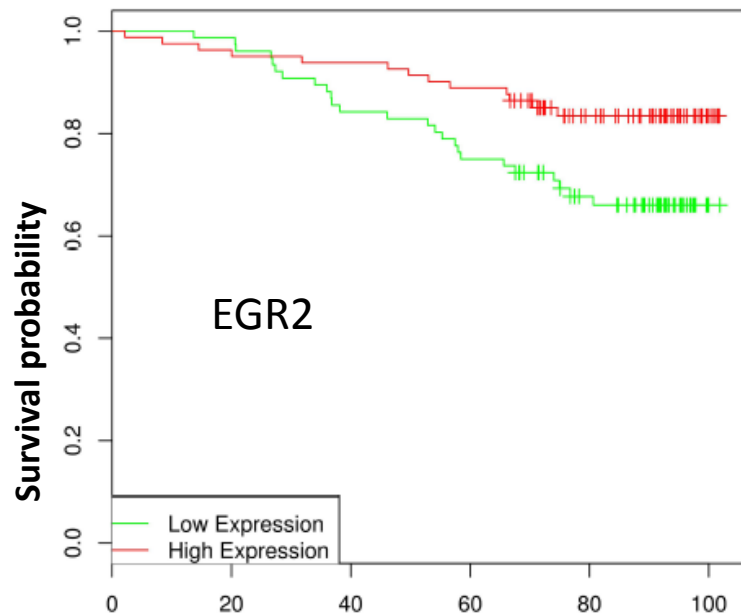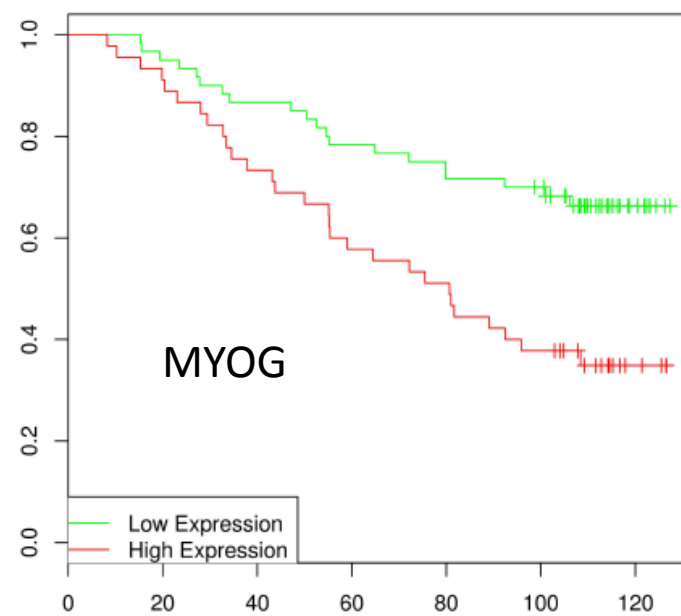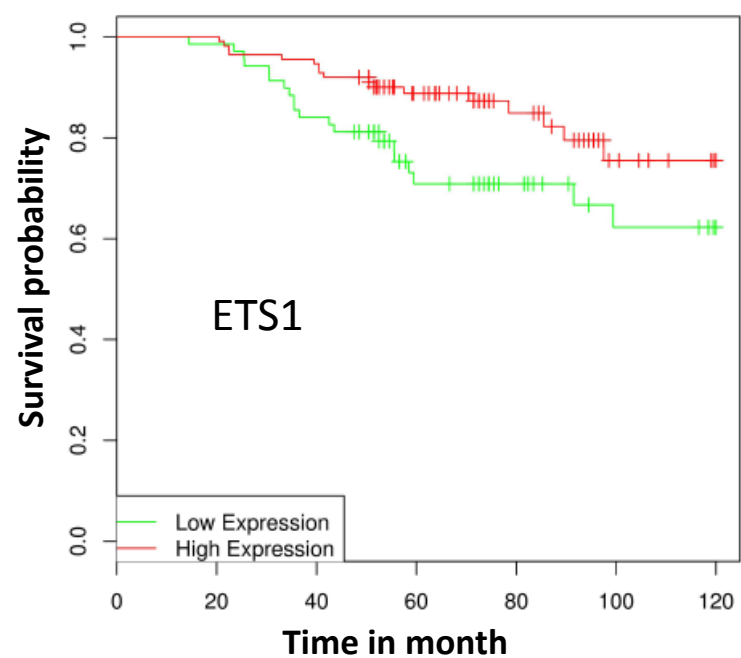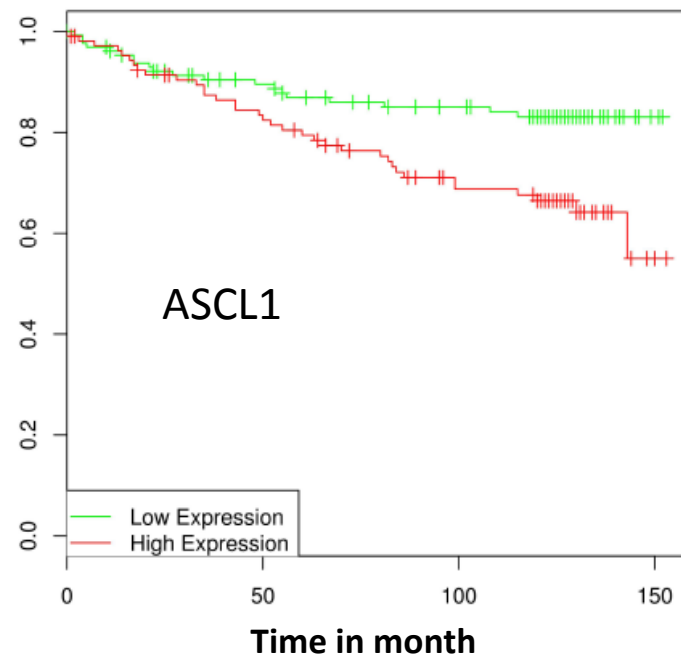

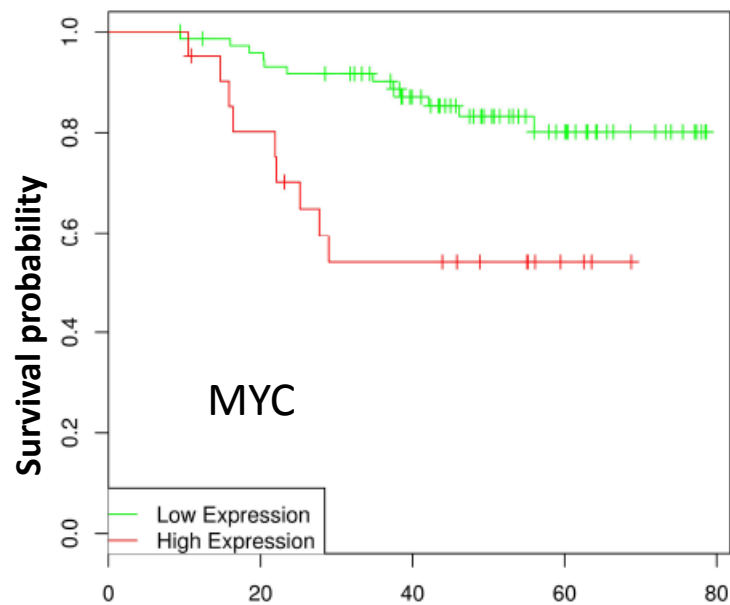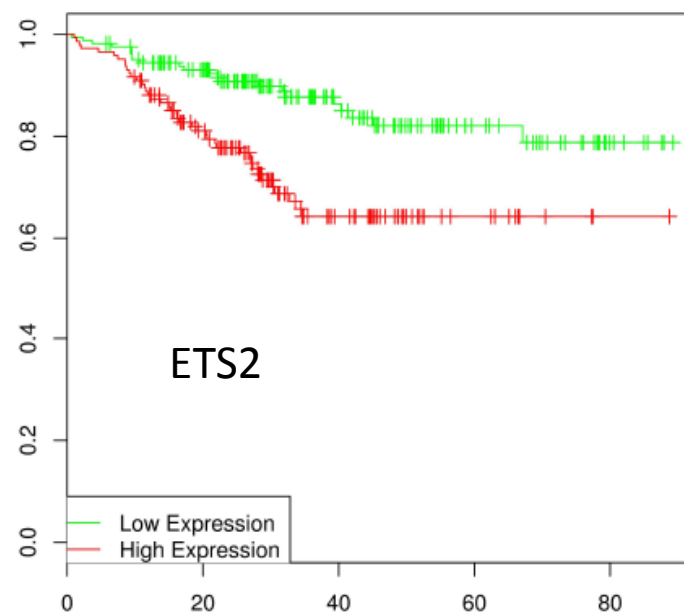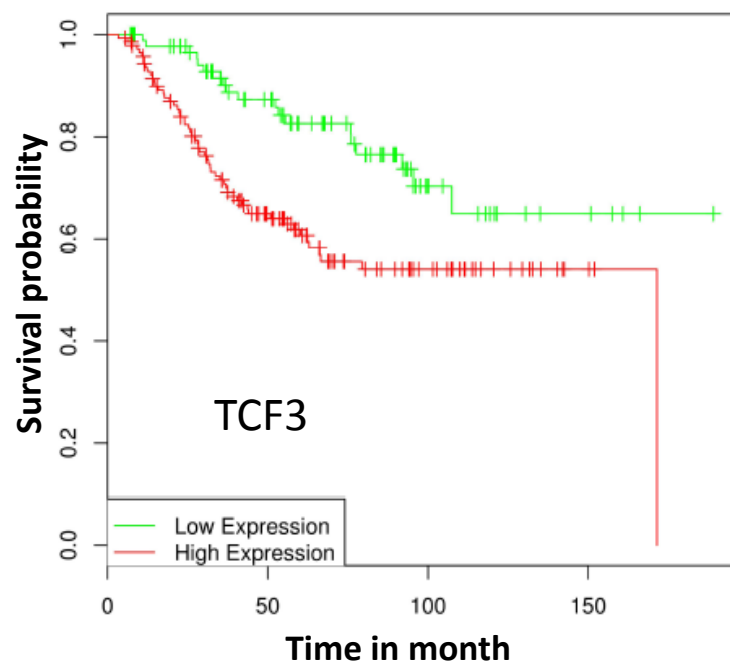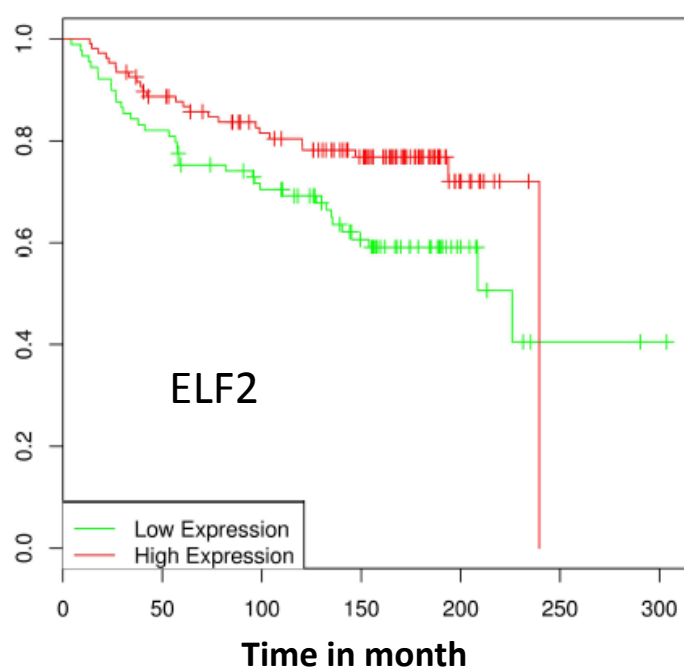

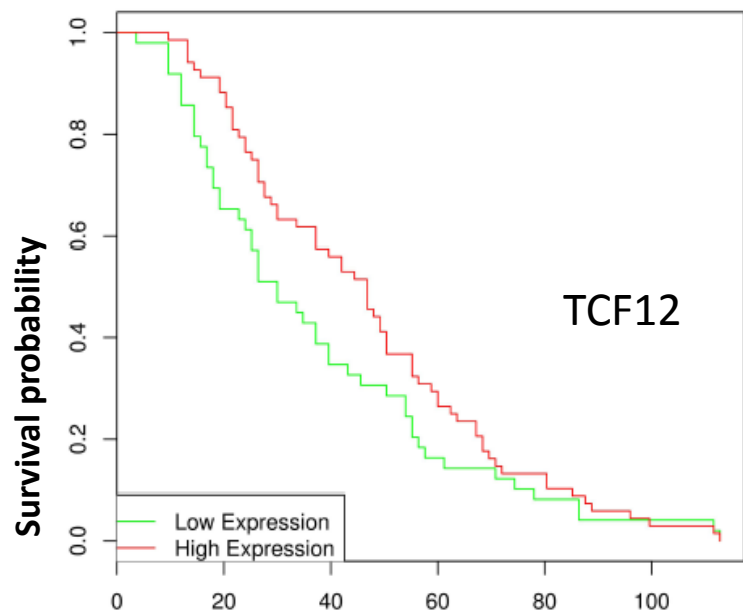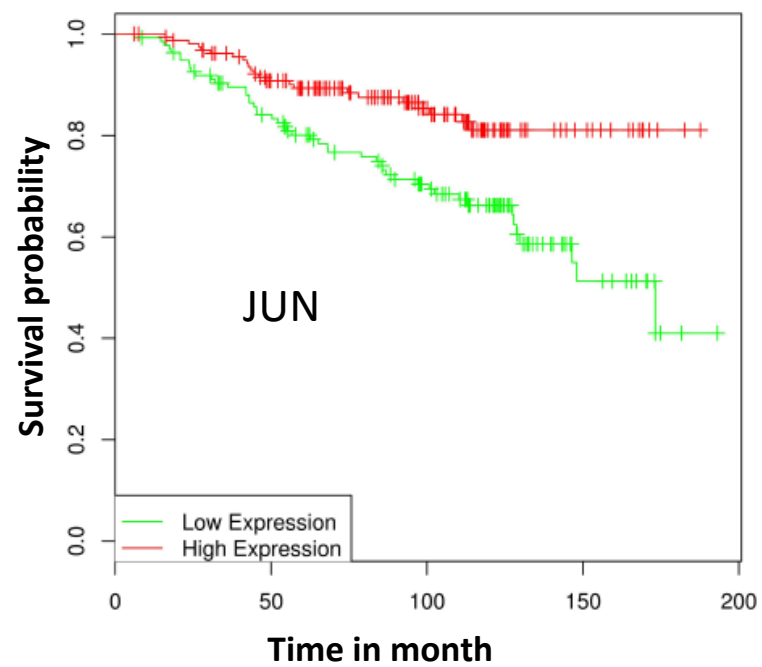

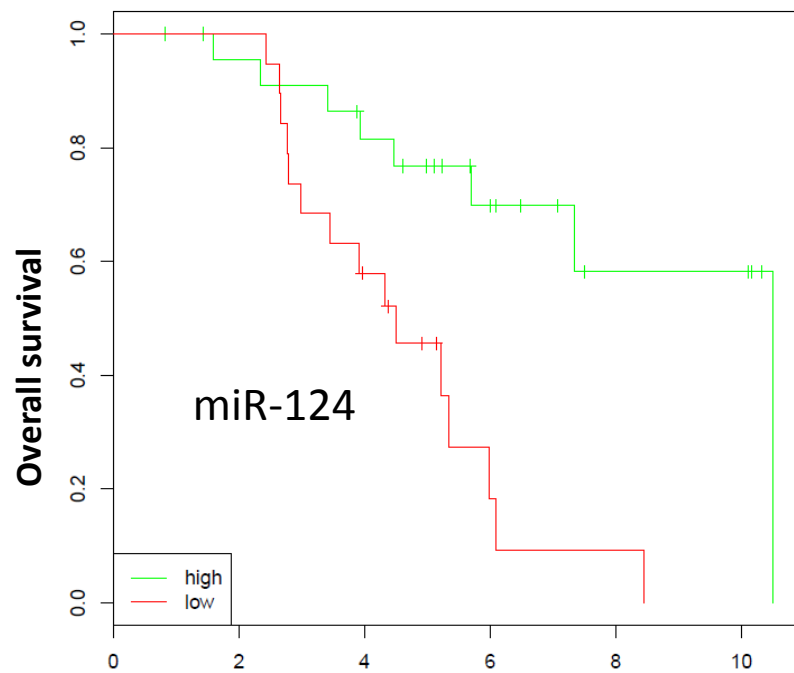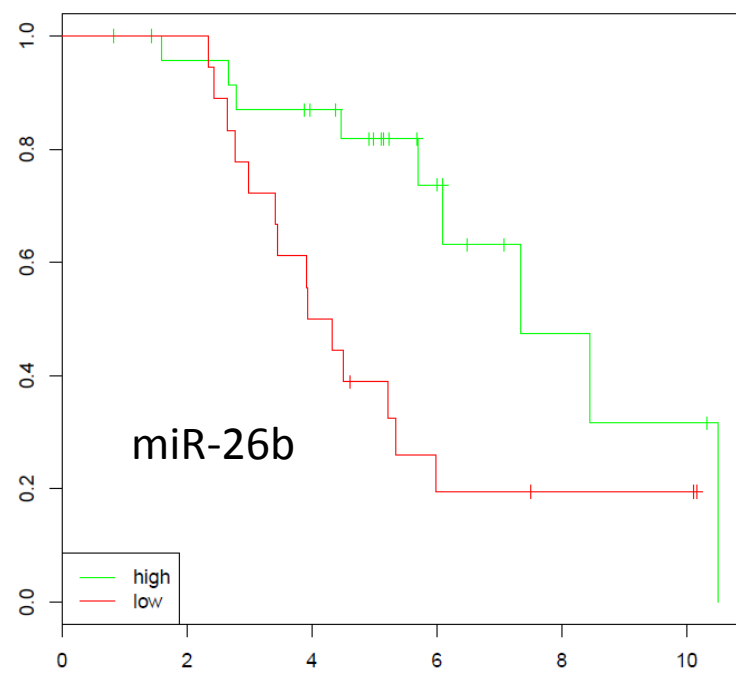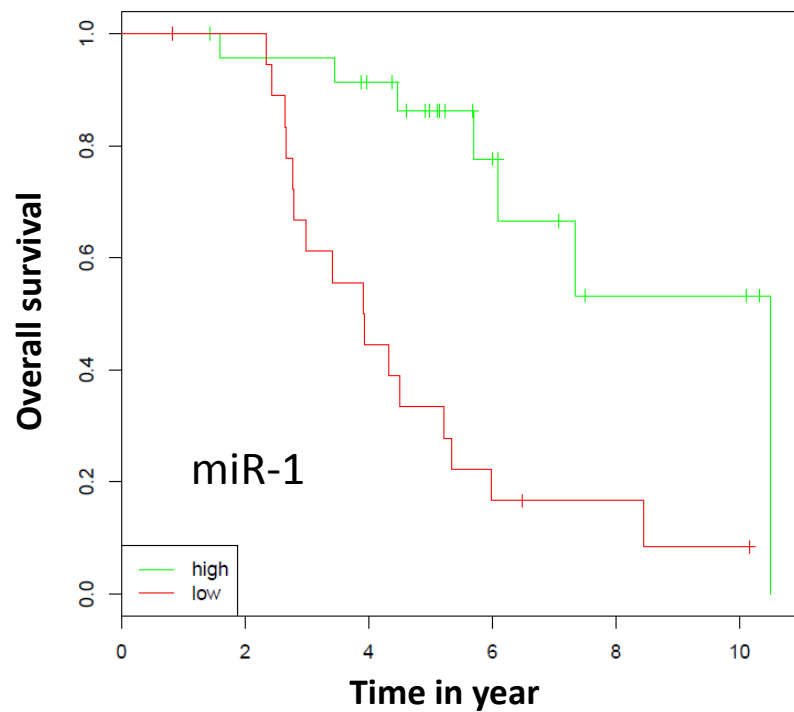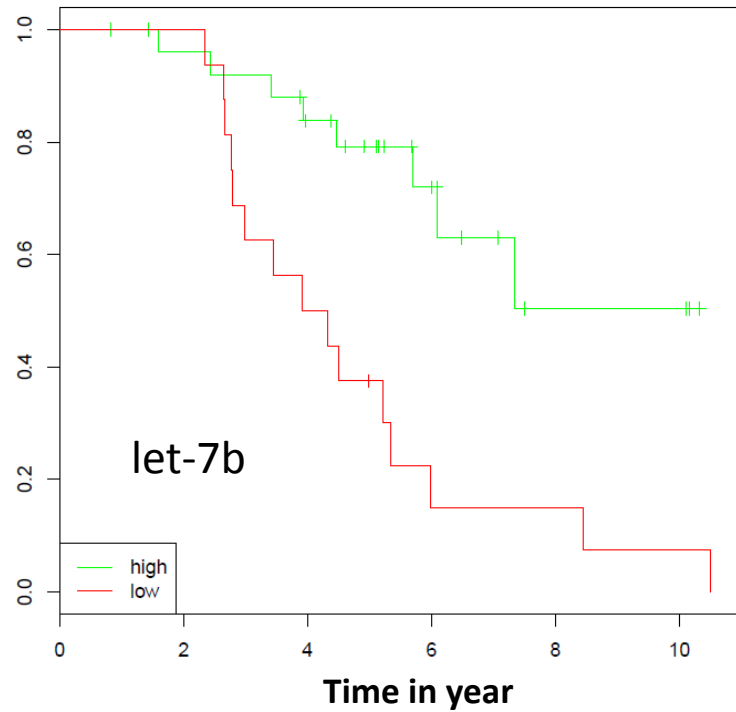

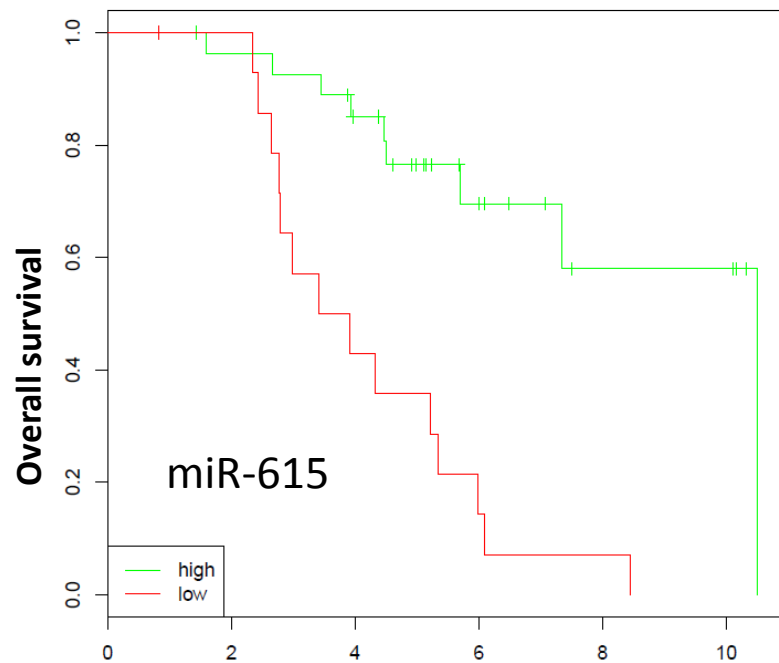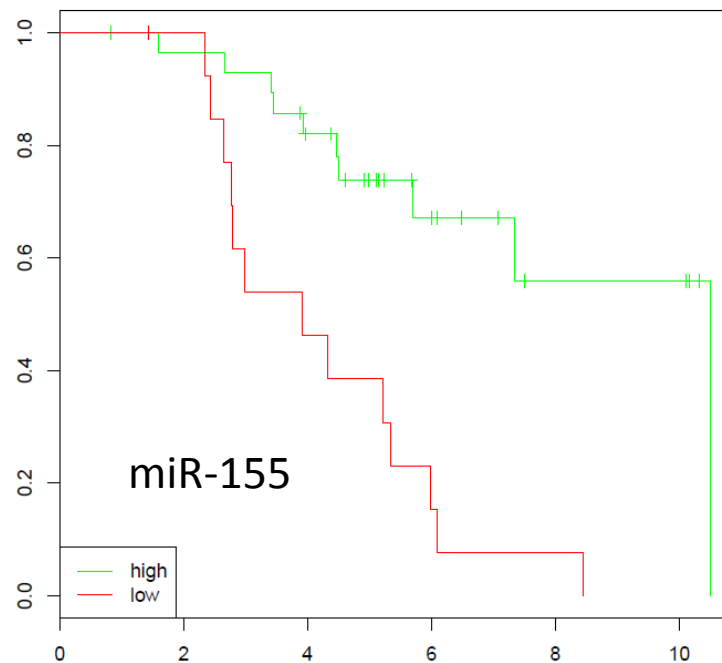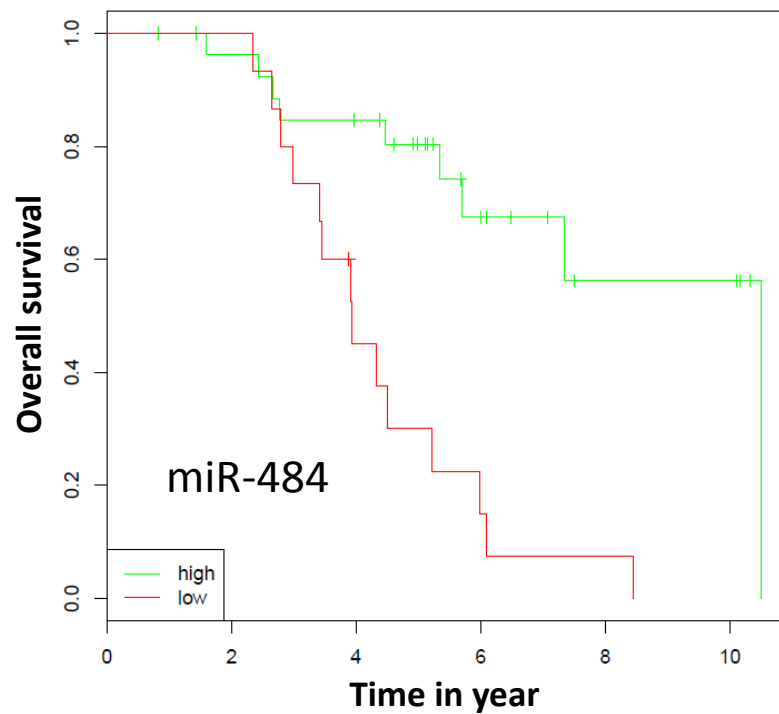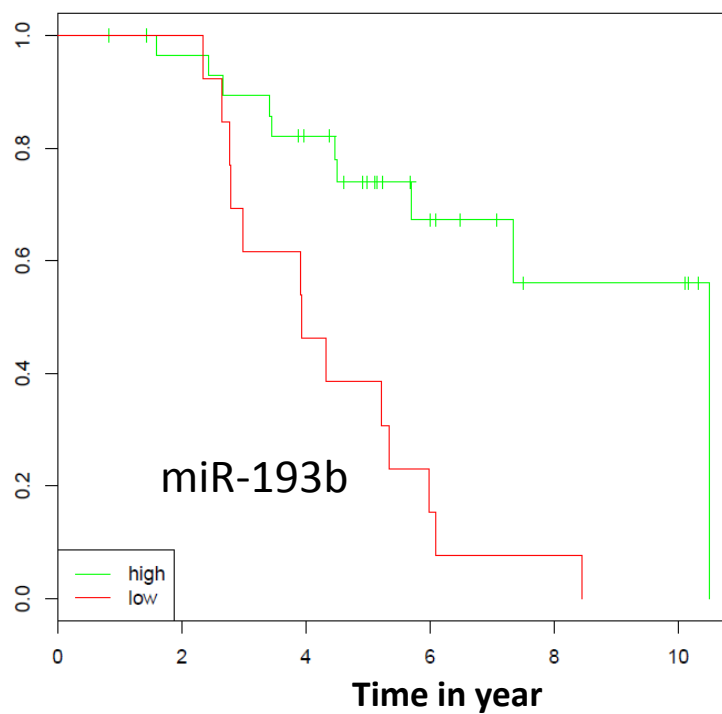

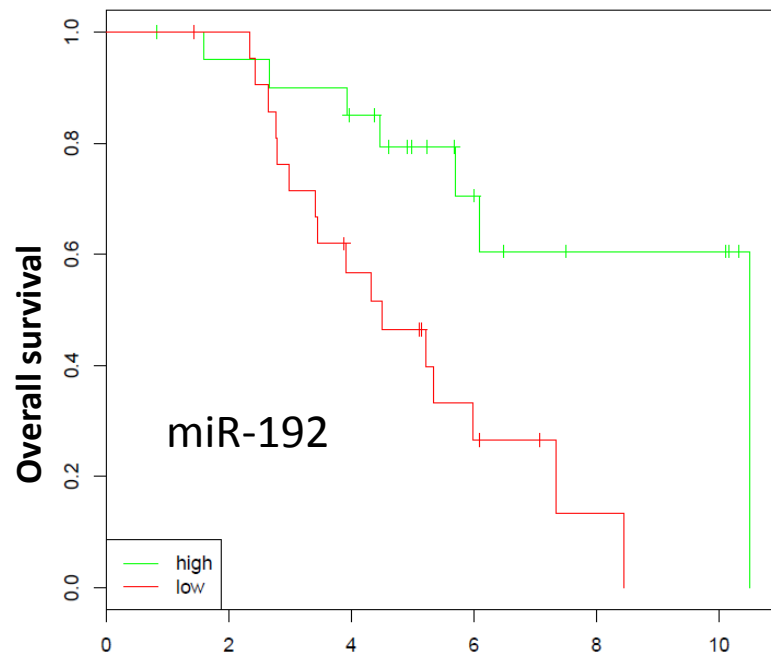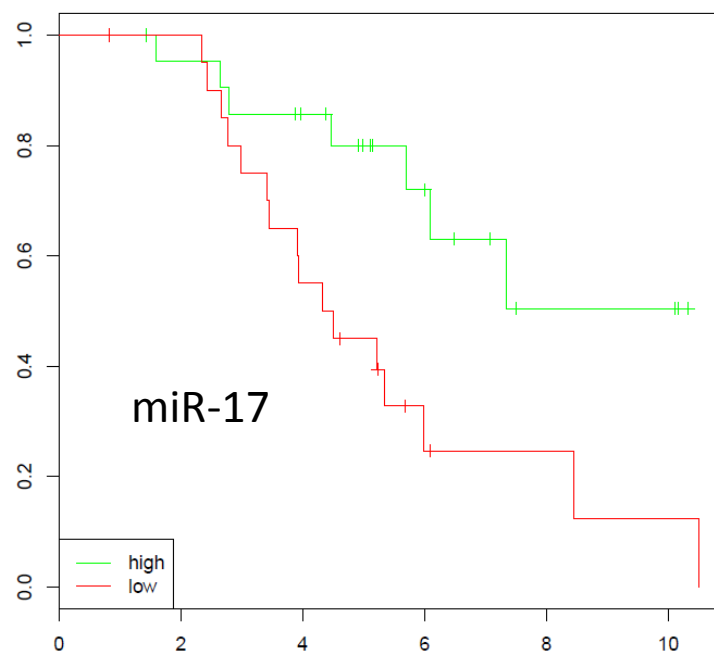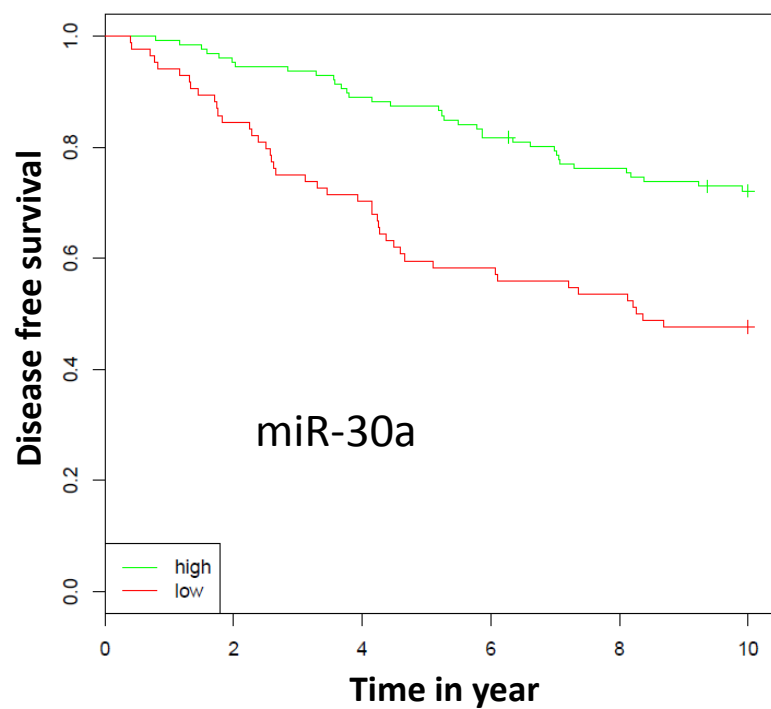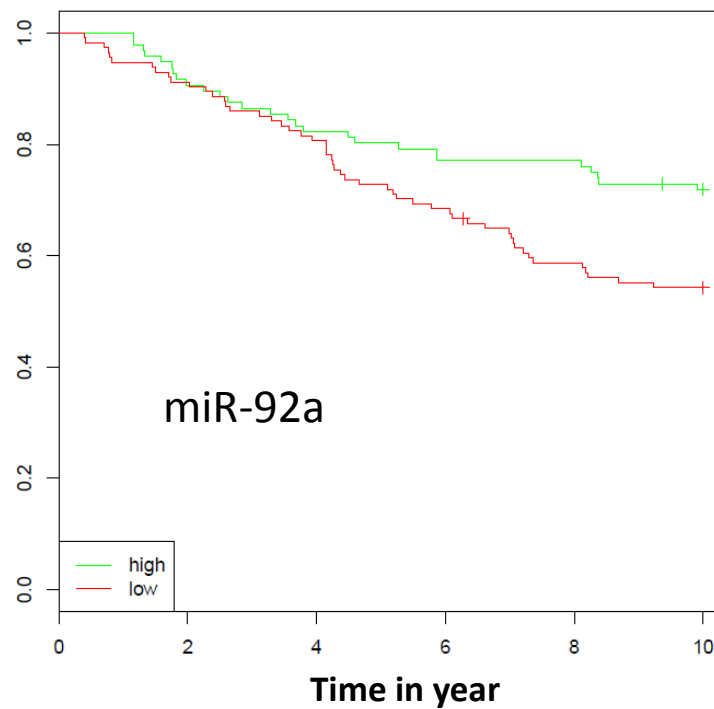

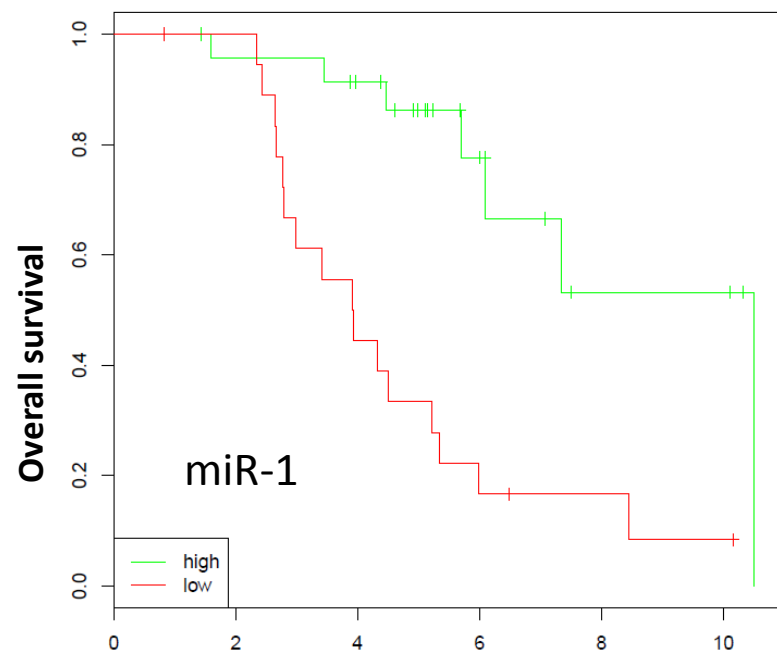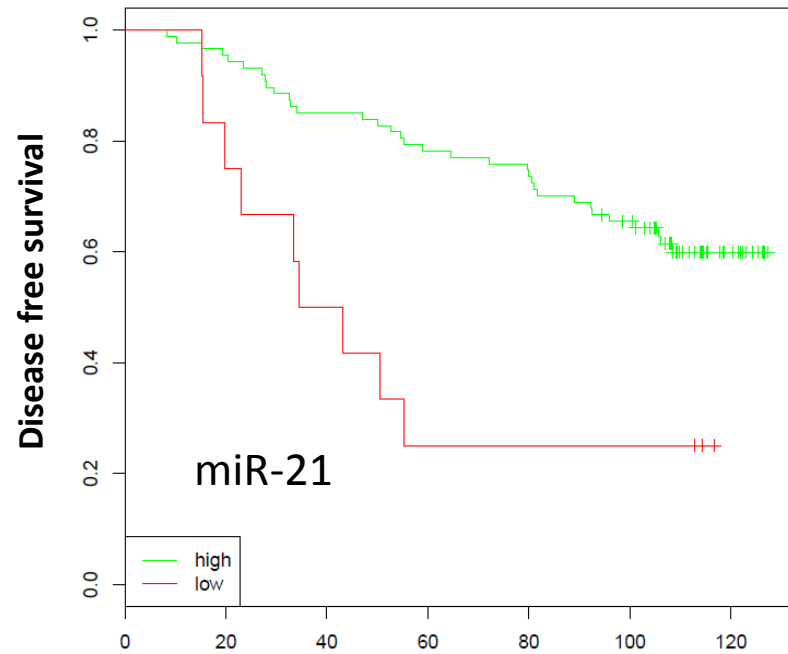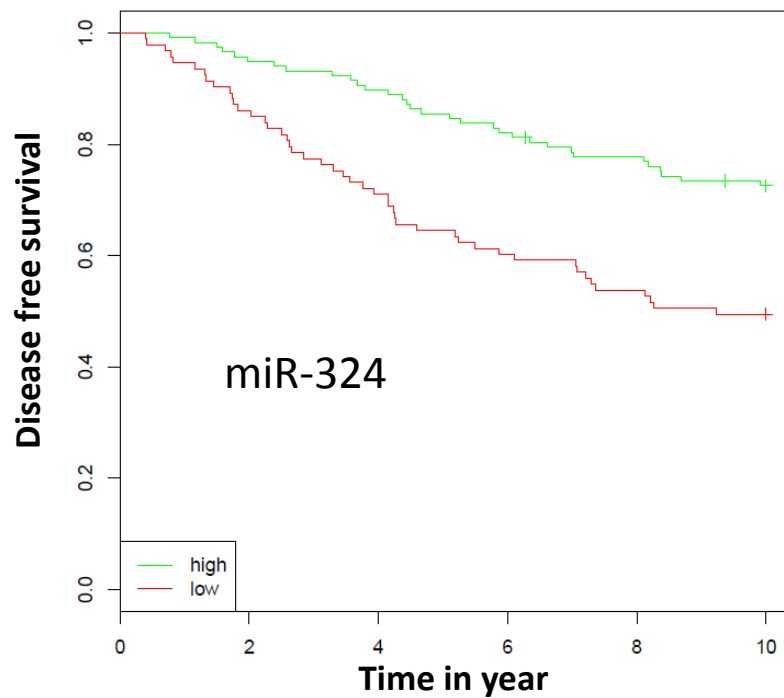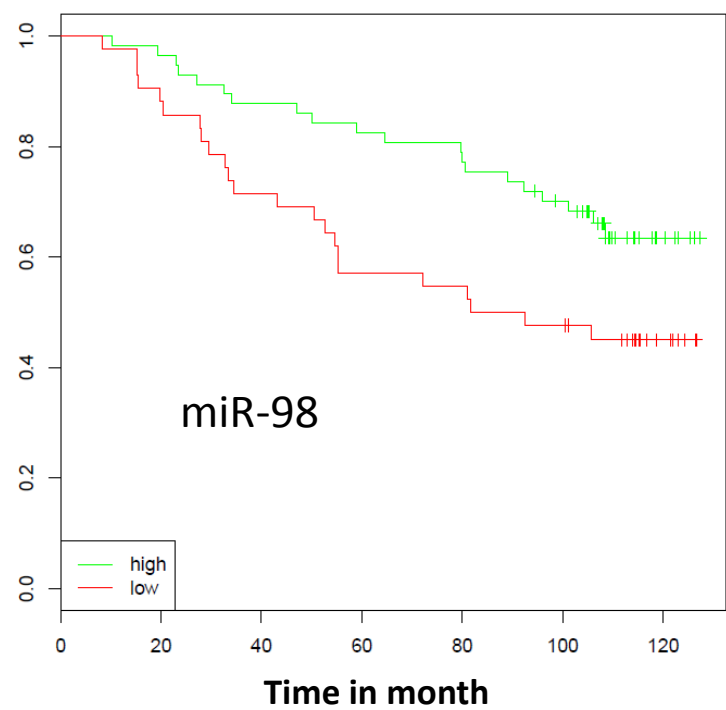

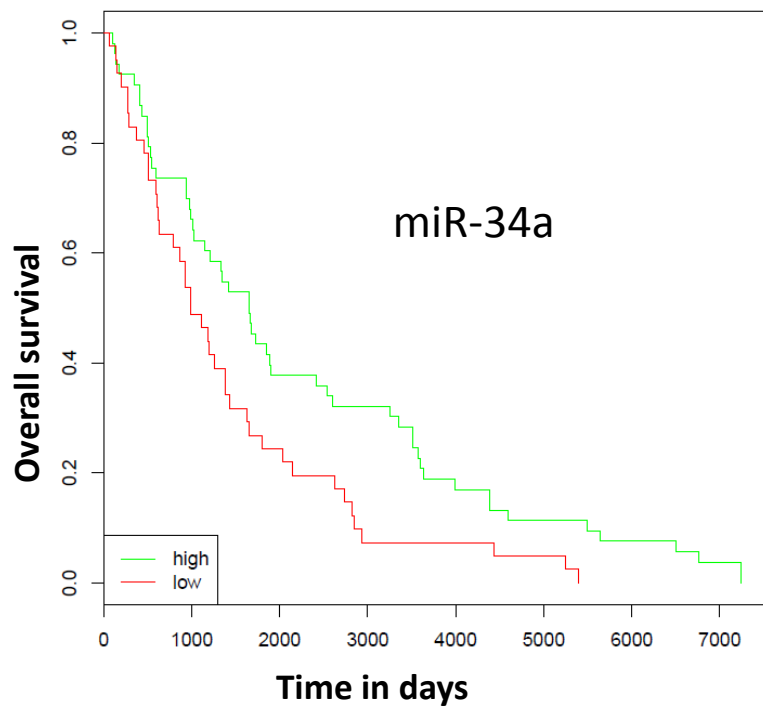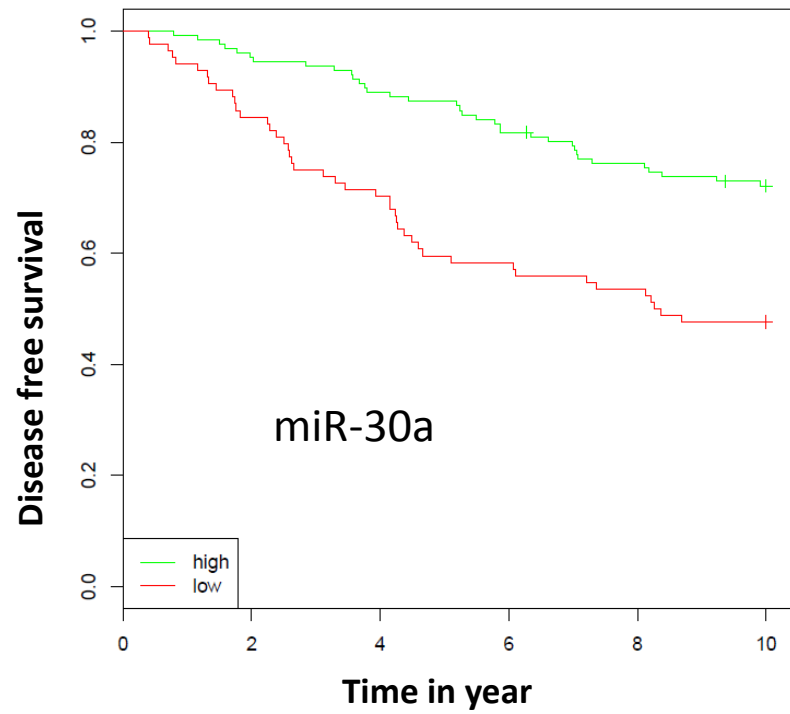

Supplement: Additional file 6: — A: The Kaplan-Meier plots for the miRNAs and TFs. B: The information about the clinical data source for survival analyses for miRNAs and TFs are provided in excel sheet. (DOC 21 kb) [file 12864_2015_2260_MOESM6_ESM.zip › Additional file6A.pdf]
