# Supplementary material for: Reconstruction of temporal activity of microRNAs from gene expression data in breast cancer cell line
Source: BMC Genomics. 2015 Dec 18;16:1077. doi: 10.1186/s12864-015-2260-3 (PMC4712512; doi:10.1186/s12864-015-2260-3)
Supplement: Additional file 7: — The complete details of the integrated procedure with original sources are provided in a pdf file. (DOC 21 kb) [file 12864_2015_2260_MOESM7_ESM.doc]

Integrated procedure

Step 1: Get the microarray or RNA sequencing (RNA-seq) gene expression data by downloading from publicly available databases or from your own experiments.

Note: Publicly available gene expression data generated either by microarrays or RNA-seq can be obtained, for instance from the following sources.

Array Express:

http://www.ebi.ac.uk/arrayexpress/

Gene Expression Omnibus:

http://www.ncbi.nlm.nih.gov/geo/

The Cancer Genome Atlas:

https://tcga-data.nci.nih.gov/tcga/findArchives.htm

Sequence Read Archive (SRA):

http://www.ncbi.nlm.nih.gov/sra

ENCODE project:

https://www.encodeproject.org/

Step 2: Do the pre-processing of the data including normalization, log transformations and conduct the significance tests. Then, filter the differentially expressed genes (DEGs) based on either P-values or fold change values or both. This pre-processing step can be easily performed using tools and packages from R/Bioconductor, MATLAB bioinformatics toolbox, SAM tools etc.

Step 3: Get the miRNA-TG, miRNA-TF, TF-TG, TF-miRNA connectivity data from publicly available databases.

Note: The connectivity information can be obtained from the following sources, for instance

miRNA-TG connectivity data:

DIANA LAB - Tarbase (Both experimental and computational prediction)

http://diana.imis.athena-innovation.gr/DianaTools/index.php?r=tarbase/index

PicTar (Computational prediction)

http://pictar.mdc-berlin.de/

TargetScan (Computational prediction)

http://www.targetscan.org/

miRTarBase (Purely experimental predictions)

http://mirtarbase.mbc.nctu.edu.tw/

TF-TG connectivity data:

TFacts

http://www.tfacts.org/

Transcriptome Browser

http://tagc.univ-mrs.fr/tbrowser/

HTRI database

http://www.lbbc.ibb.unesp.br/htri/

TRANSFAC

http://www.biobase-international.com/product/transcription-factor-binding-sites

TF-miRNA connectivity data:

TFmiR

http://service.bioinformatik.uni-saarland.de/tfmir

TransmiR

http://www.cuilab.cn/transmir

Step 4: Prepare the expression data and connectivity information into matrices and run the NCA toolbox to compute the temporal miRNA and TF activities.

Note: The NCA toolbox can be obtained from the following source.

NCA toolbox

http://www.seas.ucla.edu/~liaoj/downloads.html

Step 5: We computed the pairwise Pearson correlation coefficient (PCC) between reconstructed activity profiles of all the miRNAs and the number of common TGs between

each pair of miRNAs. We assumed a synergistic interaction between a pair of miRNAs if the Pearson correlation is greater than 0.7 and common TGs are greater than 3.

Step 6: We constructed the integrated network in the context of breast cancer. For this purpose, we started with the filtered list of DEGs and added their regulations with miRNAs and TFs. Furthermore, we added the synergistic interactions between miRNAs computed from this study and TF-miRNA interactions from the database.

Step 7: Significantly enriched biological processes and pathways are obtained by submitting the DEGs to the DAVID tool. Active sub-networks of biological processes/pathways are constructed by combining the results from DAVID tool with the integrated network. In other words, DAVID provides the information on genes involving in biological processes/pathways and their interactions with TFs and miRNAs are obtained from the built integrated network.

Note: The DAVID toolbox can be accessed from the following source.

https://david.ncifcrf.gov/

Note: All network visualizations are performed using Cytoscape software and it can be downloaded from here.

Cytoscape:

http://www.cytoscape.org/download.php
